# An artificial intelligence framework for universal landmark matching and morphometry in musculoskeletal radiography

**DOI:** 10.1007/s00330-026-12555-y

**Published:** 2026-04-22

**Authors:** Dennis Eschweiler, Eneko Cornejo Merodio, Felix Barajas Ordonez, Aleksandar Lichev, Nikol Ignatova, Marc Sebastian von der Stück, Christiane K. Kuhl, Daniel Truhn, Sven Nebelung

**Affiliations:** 1 Lab for Artificial Intelligence in Medicine, Department of Diagnostic and Interventional Radiology, University Hospital RWTH Aachen, Aachen, Germany; 2 Department of Diagnostic and Interventional Radiology, University Hospital RWTH Aachen, Aachen, Germany

**Keywords:** Artificial intelligence, Radiography, Musculoskeletal system, Anatomic landmarks

## Abstract

**Objective:**

Accurate morphometric measurements are crucial for musculoskeletal radiography, but they remain labor-intensive and prone to inter-reader variability. Current artificial intelligence-based solutions often require large annotated training datasets and narrow applications. We present and validate a training-free artificial intelligence framework that automatically derives morphometric measurements across multiple anatomies and radiographic views using universal landmark matching.

**Materials and methods:**

In this retrospective study, 600 standard radiographs of the foot, knee, and shoulder are analyzed. Additionally, a cohort of 240 challenging radiographs containing orthopedic implants was constructed to stress-test the approach. Landmarks from reference radiographs are transferred to unseen radiographs using a pre-trained generalist dense-matching method, and are then used to derive measurements in a post-processing step. The resulting measurements were compared with manual annotations and measurements by two radiologists.

**Results:**

Mean landmark matching error is 2.68 ± 2.70 mm using a single reference radiograph and improves to 2.15 ± 2.38 mm with 40 reference radiographs. Measurement accuracy ranges from 1.81° (I–II metatarsal angle) to 8.65° (congruence angle). Increasing the number of reference images improved measurement accuracy, and mostly approached inter-reader agreement. Performance is mixed on the challenging cohort, demonstrating the limitations and strengths of the approach.

**Conclusions:**

This anatomy-agnostic framework enables training-free morphometry across multiple regions, with measurement-dependent performance often comparable to inter-reader agreement. Challenging cases highlight specific limitations, motivating the use of quality control and reference-set tuning for deployment. Its minimal setup enables rapid adaptation to new anatomies and measurements, and clinically practical runtimes require GPU inference.

**Key Points:**

***Question*** *Can a generalist artificial intelligence framework be used to accurately and automatically perform morphometric measurements across different musculoskeletal radiographs without anatomy-specific training?*

***Findings**** The training-free approach achieved performance that approaches expert-level agreement for most measurements, while highlighting measurement-specific limitations in challenging cases. Multiple reference radiographs improved results*.

***Clinical relevance**** This approach automates repetitive morphometric measurements that are prone to inter-reader variability, reducing manual workload while providing reproducible results that can approach expert radiologist performance. Its adaptability and minimal setup enable integration into routine workflows*.

**Graphical Abstract:**

**Figure:**
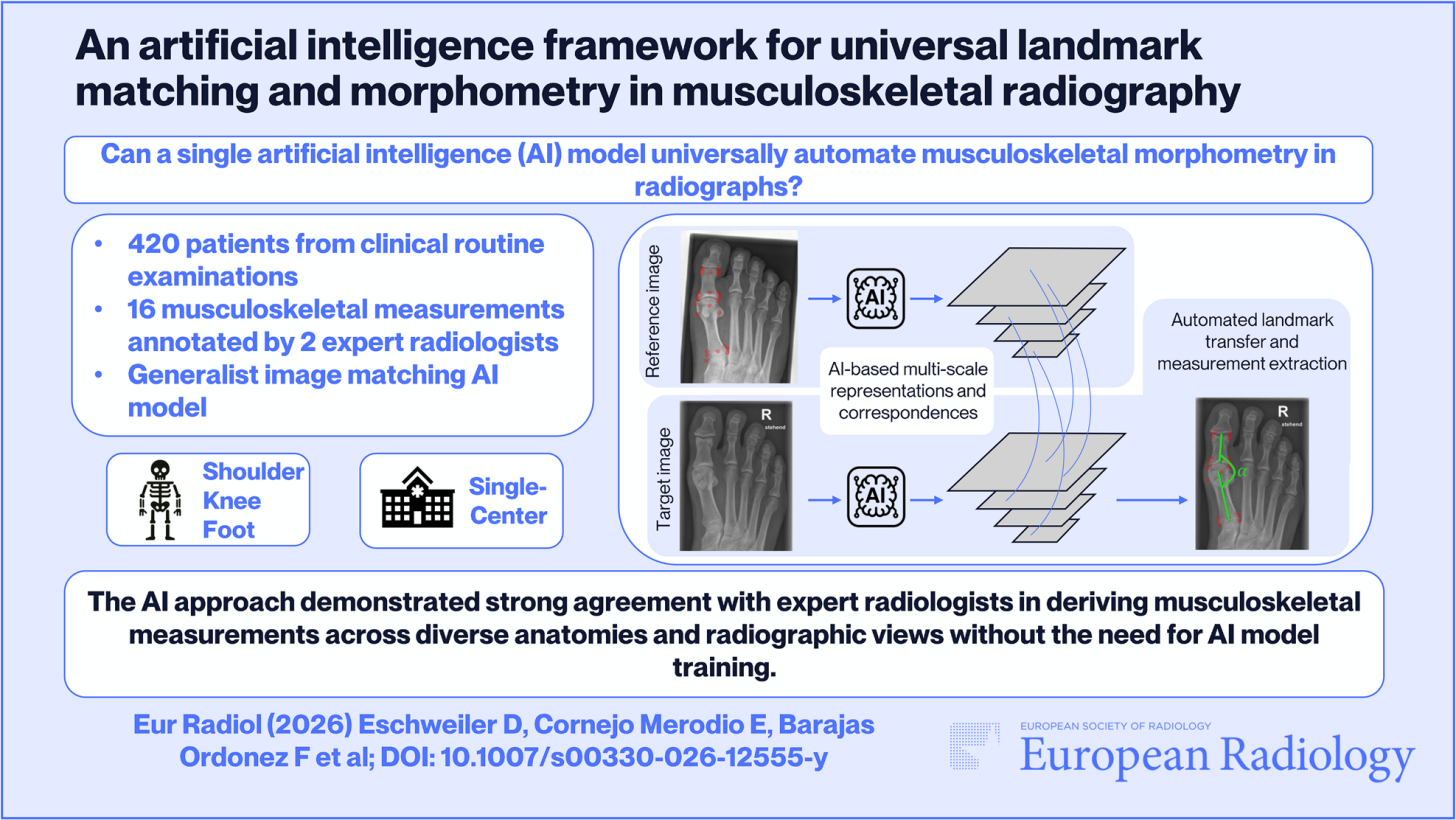

## Introduction

Quantitative assessment of radiographs is essential for diagnosing and monitoring musculoskeletal conditions, including deformities, joint-space narrowing, and anatomic malalignment. Such assessments hinge on the precise localization of specific anatomic landmarks, which vary by body region and clinical question. Currently, these landmarks are still placed manually by radiologists, a labor-intensive step that is vulnerable to inter-reader variability and fatigue-related errors [1–3]. Although modern Picture Archiving and Communication System (PACS) workstations provide measurement tools, they nevertheless depend on correct manual landmark identification and extensive user interaction.

Artificial intelligence (AI) solutions have the potential to mitigate these limitations by standardizing and accelerating routine measurements [4]. AI models have been developed for fully automated analysis of hip [5–8], lower-extremity [9–14], shoulder [15–17], and foot radiographs [18–20]. Most of these pipelines rely on segmentation networks that outline bone contours before deriving measurements, or require dedicated training datasets. Unfortunately, segmentation accuracy can degrade with altered morphotypes, hardware, or overlapping structures. When this occurs, errors propagate to the subsequent measurement steps. Fully-annotated and specific training datasets, frequently with pixel-accurate annotations, make it difficult to extend to other anatomic regions. Moreover, such datasets are scarce and typically small, with few examples of altered morphotypes, rare pathologies, or metallic implants, ultimately limiting model generalizability in precisely these challenging but clinically relevant scenarios [21].

**Fig. 1:**
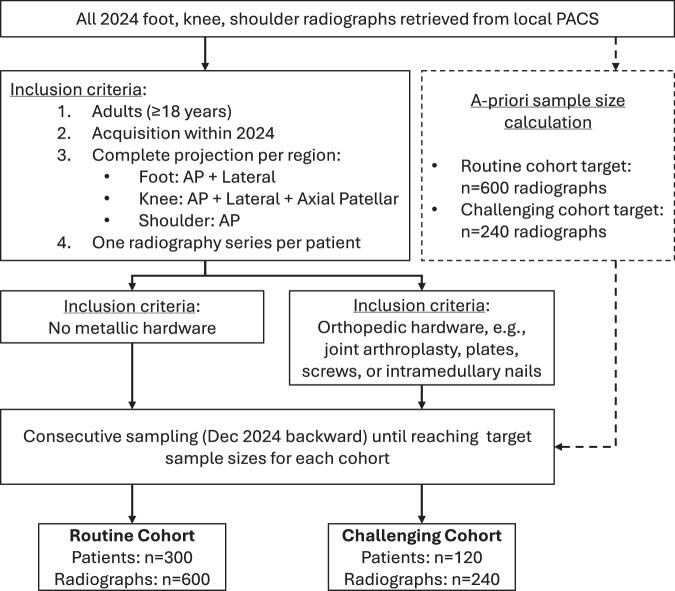
Data selection and cohort construction. Radiographs from 2024 were retrieved from the institutional PACS and screened using predefined inclusion criteria. A priori precision-based calculations defined the required sample sizes, and radiographs were then consecutively selected backward from December 2024 until these targets were reached. Eligible cases were assigned to either the routine cohort without implants or the challenging cohort with orthopedic hardware

We aim to address these limitations by proposing a minimal-setup, anatomy-agnostic AI framework that formulates landmark localization as a dense matching task between one or more reference radiographs and unseen target radiographs (Fig. 1). Although some manual effort is required to annotate the reference radiograph(s), it is negligible compared to the effort needed for more traditional approaches that require large datasets and extended training times. By leveraging a fully pretrained matching backbone [22] and incorporating redundancy in both landmark definitions and reference selection, we hypothesize that the dense matching-based method can be readily deployed to any anatomic region without further training.

Our objectives were (i) to develop a universal landmark-matching framework that automatically derives standard morphometric measurements from foot, knee, and shoulder radiographs, (ii) to quantitatively evaluate landmark and measurement accuracy across multiple projections, increasing numbers of reference images, and challenging cases, and (iii) to benchmark the framework against radiologists’ inter-reader agreement. Consequently, we hypothesize that (i) the dense-matching approach will localize landmarks with a mean absolute error (MAE) of less than 3 mm, independent of anatomic region, (ii) inter-method variability will approach inter-reader variability for the majority of morphometric measurements, and (iii) the framework will remain functional in the presence of orthopedic implants, albeit with measurement-dependent variation in accuracy.

## Materials and methods

### Ethical approval and study design

This research complies with all relevant ethical regulations. Ethical approval for this single-center, retrospective, intra-individual comparative study was granted by the Institutional Review Board of the medical faculty of University Hospital RWTH Aachen under EK24-148. We selected the foot, knee, and shoulder as anatomic regions, and there was no significant overlap of patients with prior work. Radiographs were retrieved from the institutional PACS (Vue PACS, Philips) and screened with four prespecified criteria (Fig. 1):

(i) Adults only: patient age ≥ 18 years;
(ii) Acquisition time period 2024: radiography series acquired between January 2024 and December 2024 in either inpatient or outpatient settings;
(iii) Complete projections per anatomic region: foot—anteroposterior (AP) and lateral; knee—AP and lateral and axial patellar; shoulder—AP only;
(iv) One radiography series per patient to avoid redundancy; when multiple studies met the above criteria, the earliest study from 2024 was included.

For the shoulder, the AP view was selected to streamline data preparation, as the wide variation in arm rotation, beam angle, and patient positioning for additional views (axillary-lateral, scapular-Y) would require generating separate reference sets. This choice was made for study feasibility and does not reflect any inherent restriction of the proposed method. Notably, no exclusion criteria based on image quality were applied, and radiographs were analyzed as acquired from PACS, thereby reflecting routine clinical variability.

After applying the selection criteria, we divided the study population into two cohorts. The ‘routine cohort’ comprised 600 radiographs from 300 consecutive adult patients who met all prespecified criteria and showed no metallic implants. Patients were middle-aged on average (48 ± 19 years; 47% [*n* = 141] men) and largely reflected the spectrum of cases seen in daily practice. To stress-test the AI framework, we assembled a ‘challenging cohort’ of 120 patients (240 radiographs) with orthopedic hardware, e.g., joint arthroplasty, plates, screws, or intramedullary nails. Patients were older (56 ± 16 years; 44% [*n* = 53] men). Demographic details of the two cohorts are provided in Table 1. All selected radiographs (*n* = 840) were included in the final evaluation, as the framework did not require model training.

**Table 1:** Demographic characteristics of the study cohorts

	Anatomic region	Patients (*n*)	Radiographs (*n*)	Male (*n* [%])	Female (*n* [%])	Age [y]	Inclusion specifications
Routine cohort	Foot	100	200	38 [38%]	62 [62%]	49 (± 16)	No orthopedic hardware^*^
	Knee	100	300	48 [48%]	52 [52%]	46 (± 20)	
	Shoulder	100	100	55 [55%]	45 [45%]	48 (± 19)	
Challenging cohort	Foot	40	80	21 [53%]	19 [47%]	57 (± 19)	Orthopedic hardware^*^
	Knee	40	120	16 [40%]	24 [60%]	59 (± 16)	
	Shoulder	40	40	16 [40%]	24 [60%]	53 (± 13)	

Patient age is indicated as mean (± standard deviation)

^*^ Orthopedic hardware was defined as arthroplasties, plates, screws, or intramedullary nails

### Statistical justification of minimum sample size

We planned the sample size to estimate the MAE per projection with prespecified precision. Using the normal approximation for a 95% confidence interval (CI) of a mean difference, the required *n* is *n* = (1.96 × σ/h)², where σ is the anticipated standard deviation (SD) of errors and h is the desired half-width of the CI. Given the wide range of included anatomies, projections, and metrics, deriving inter-observer variability metrics from the literature is challenging. Using published inter-observer variability as inspiration [23–25], we intentionally set the framework parameters to be conservative.

For the routine cohort, we anticipated σ ≈ 2.5° for angles and σ ≈ 1.0 mm for distances. Targeting *h* = 0.5° and *h* = 0.25 mm gave *n*_angle_ ≈ 96 and *n*_distance_ ≈ 61 per projection. We adopted the conservative value (≈100 per projection). Across the six projections (foot AP/lateral, knee AP/lateral/patellar axial, and shoulder AP), this estimation yielded ~600 radiographs.

For the **c**hallenging cohort, we expected higher observer spread/variability (σ ≈ 4.5°, σ ≈ 1.4 mm) and accepted slightly wider precision (*h* = 1.5°, *h* = 0.5 mm). Analogously, the calculation yielded *n*_angle_ ≈ 35 and *n*_distance_ ≈ 31 per projection. By rounding up to ~40 per projection, i.e., ~240 radiographs across six projections, we aimed to increase the safety margin.

### Landmark and measurement definition

For each anatomic region and projection, we selected standard, clinically validated morphometric measurements based on guidelines and prior literature [14–20]. All considered measurements per anatomic region are listed in Table 2. Two radiologists (E.C.M. and F.B.O.; 1 and 5 years of experience) performed manual measurements in PACS, blinded to the original report and to each other’s results. Landmark definitions used to derive those measurements (Supplementary Figs. S1–S3) were established by E.C.M. together with a senior MSK radiologist (S.N.; 12 years). Reference landmark annotation was performed by two pre-graduate medical students (A.L. and N.I.) under close supervision (E.C.M.) using 3D Slicer (v5.8.1, Surgical Planning Laboratory [26]). Annotations were quality-checked by E.C.M. and S.N. Importantly, F.B.O. (R2) was not involved in landmark definition, student supervision, or quality control and served as an independent reader for the performance evaluation.

**Table 2:** Selected sets of morphometric measurements for each anatomic region and radiographic view

Anatomic region	Projection	Landmarks (*n*)	Morphometric measurements
Foot	AP	68	Hallux-Valgus angle, I–II Intermetatarsal angle, I–V Intermetatarsal angle
	Lateral	23	Medial arch angle, Meary’s angle, calcaneal inclination
Knee	AP	12	Medial joint space, lateral joint space
	Lateral	6	Insall–Salvati ratio, Caton–Deschamps index
	Axial patellar	4	Sulcus angle, congruence angle
Shoulder	AP	19	Critical shoulder angle, lateral acromion angle, acromial index, acromiohumeral interval

For each projection, the total number of landmarks is reported, whereas individual measurements are derived from a subset of these landmarks

*AP* anteroposterior

### Automated landmark matching

**Fig. 2:**
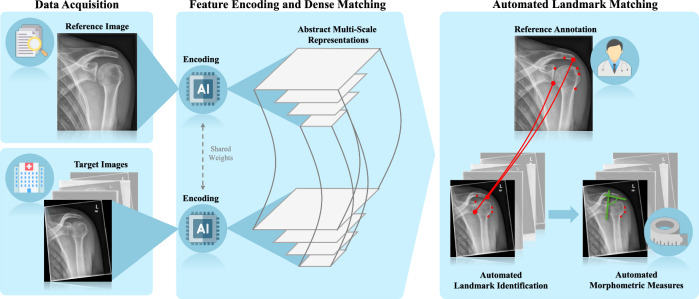
Workflow of the proposed anatomy-agnostic landmark-matching pipeline. From left to right: a manually annotated *reference* radiograph and unseen target radiographs enter the pipeline. Both images are passed (using shared weights) through a pretrained hybrid encoder [22] (DINOv2 transformer for coarse context followed by a CNN feature pyramid) to obtain a multi-scale, abstract representation, and dense correspondences are then established between the two feature stacks. Each landmark defined in the reference image is automatically projected onto the target image via the dense-matching field, enabling the calculation of morphometric measurements without any anatomy-specific retraining

We used a pretrained dense-matching framework [22] that combines a “Distillation of Knowledge with No Labels” (DINOv2) transformer-encoder [27] for coarse, low-resolution context (14 × 14-pixel patches) with a convolutional neural network (CNN)-based feature pyramid [28] for fine, high-resolution detail. The framework yields a dense correspondence field between a reference and a target radiograph, allowing any landmark defined in the reference to be transferred instantly to the target (Fig. 2). Pixel intensities were z-normalized to ImageNet statistics [29], and images were rescaled to 560 × 560 pixels to reduce computational demand during encoding. Matches were later upsampled to the original resolution for measurement and evaluation, enabling robust global matching while remaining resilient to image impurities and inconsistencies.

Preliminary tests showed that matching performance depended on the chosen reference image. Therefore, within the routine cohort, we matched each patient’s landmarks to all others and ranked all patients according to the mean Euclidean distance across all pairings, from smallest to largest. The reference image was then selected automatically from the top-ranked patient. Using multiple reference images may reduce uncertainty, especially in regions with superpositions, and enhance matching reliability. To leverage multiple references, we selected the top-k patients, each generating an independent landmark prediction on the target image. The final landmark coordinates were obtained by combining these predictions through their geometric median. To avoid cohort-dependent reference selection and ensure a consistent operating point, reference images were selected only from the routine cohort (per projection) and used as a reference set for landmark matching in both the routine and challenging cohorts. All experiments were performed at identical reference-set sizes (k = 1, 5, 10, 20, 40).

To investigate the internal representations learned by the framework, we performed a post-hoc visualization using the feature pyramids generated during the matching process. For a given query point in the reference image, feature vectors from all levels of the pyramid were concatenated to form a single multi-scale representation. This representation was compared to the feature vectors of all positions in the target image, with similarity quantified using Euclidean distance. The resulting heatmaps highlight regions in the target image that are most similar to the reference query point, providing insight into the spatial correspondences identified by the model and revealing the semantic information encoded in its internal features.

### Time demand

Time demand for running the landmark matching procedure was determined on a workstation with a dedicated graphics processing unit (Intel i7-10700K, 128 GB memory, NVIDIA GeForce RTX 3090 24 GB) and, to assess lower limit performance, on a consumer-grade notebook (Intel Ultra 7 165U Processor, 32 GB memory, no dedicated GPU). The latter setup is not intended to reflect realistic deployment conditions, but demonstrates that the method can still run on minimal hardware.

### Statistical analysis

Statistical analysis was performed by DE, ECM, and SN in Python 3.9 using scipy 1.13.1. To assess the landmark error, the mean Euclidean distance and its SD between the manual references and the predicted landmarks were calculated. To assess the measurement error, the MAE and SD between automated values (derived from predicted landmarks) and both radiologists’ manually measured values were calculated. Inter-rater and inter-method agreement were assessed pairwise using MAE, Bland–Altman bias with 95% limits of agreement, and intraclass correlation coefficients (ICC). ICCs were computed as a two-way random-effects model for single measurements with absolute agreement (ICC(2,1)) and 95% CIs.

## Results

### Patient cohorts

This study included two cohorts, referred to as the “routine” and the “challenging” cohorts. Both cohorts contained complete radiographic projections for three anatomic regions: foot (AP and lateral views), knee (AP, lateral, and axial patellar), and shoulder (AP only). The routine cohort consisted of 600 radiographs from 300 consecutive adult patients without metallic implants. The mean age was 48 ± 19 years, and 47% (*n* = 141) were men. This group broadly represented the spectrum of cases encountered in daily clinical practice. To evaluate the framework under more difficult conditions, an additional challenging cohort was assembled, comprising 240 radiographs from 120 patients with orthopedic hardware, including joint arthroplasties, plates, screws, and intramedullary nails. The mean age was 56 ± 16 years, and 44% (*n* = 53) were men.

### Accuracy and precision of landmark matching and derived measurements on the routine cohort

**Fig. 3:**
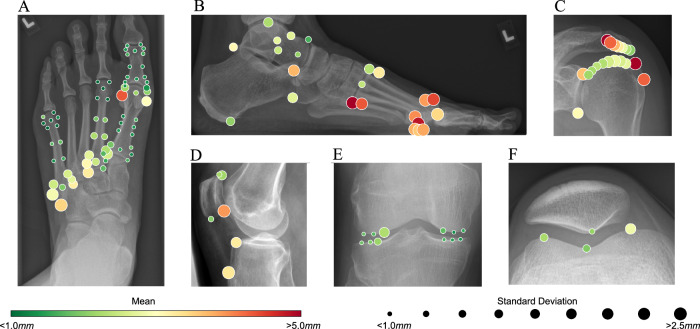
Spatial distribution of landmark-matching errors across all projections with one reference image. Panels show the landmark-matching accuracy in terms of the mean Euclidean distance between predicted and manually annotated landmarks (color-coded) and precision in terms of its SD (dot size) for every annotated landmark in the routine cohort. Regions of consistently high precision are highlighted by small green dots, while regions where matching is less stable are highlighted by orange-to-red larger dots, most notably around the acromion, inferior patella, and the metatarsals. The landmarks were used to derive the following morphometric measurements: (**A**) Foot AP: Hallux Valgus angle, I–II metatarsal angle, I–V metatarsal angle, (**B**) foot lateral: medial arch angle, Maery’s angle, calcaneal inclination, (**C**) shoulder AP: critical shoulder angle, lateral acromion angle, acromial index, acromiohumeral interval, (**D**) knee lateral: Insall–Salvati ratio, Caton–Deschamp Index, (**E**) knee AP: medial and lateral joint spaces, and (**F**) knee patellar axial: sulcus angle and congruence angle. More detailed descriptions of the morphometric measurements are presented in Supplemental Figs. S1–S3

Across all three anatomic regions and projections, the mean landmark error was 2.68 ± 2.70 mm (foot: 2.30 ± 2.32 mm; knee: 3.13 ± 3.09 mm; shoulder: 3.16 ± 2.77 mm), with variable accuracy and precision. Figure 3 illustrates the spatial distribution of landmark-matching errors for a single reference image. Landmark matching accuracy was also evaluated in distinct subsets, each comprising only the landmarks used to derive a specific morphometric measurement. Average errors for these measurement-specific subsets were largely low (i.e., lower than 3 mm; Supplementary Table S1). Reduced accuracy was observed at the metatarsals, the inferior pole of the patella, and the acromion, resulting in relatively high errors for related measurements-specific landmark subsets, such as Meary’s angle (3.29 ± 2.64 mm), the Insall–Salvati ratio (3.90 ± 3.25 mm), and the Acromial index (4.01 ± 3.35 mm).

**Fig. 4:**
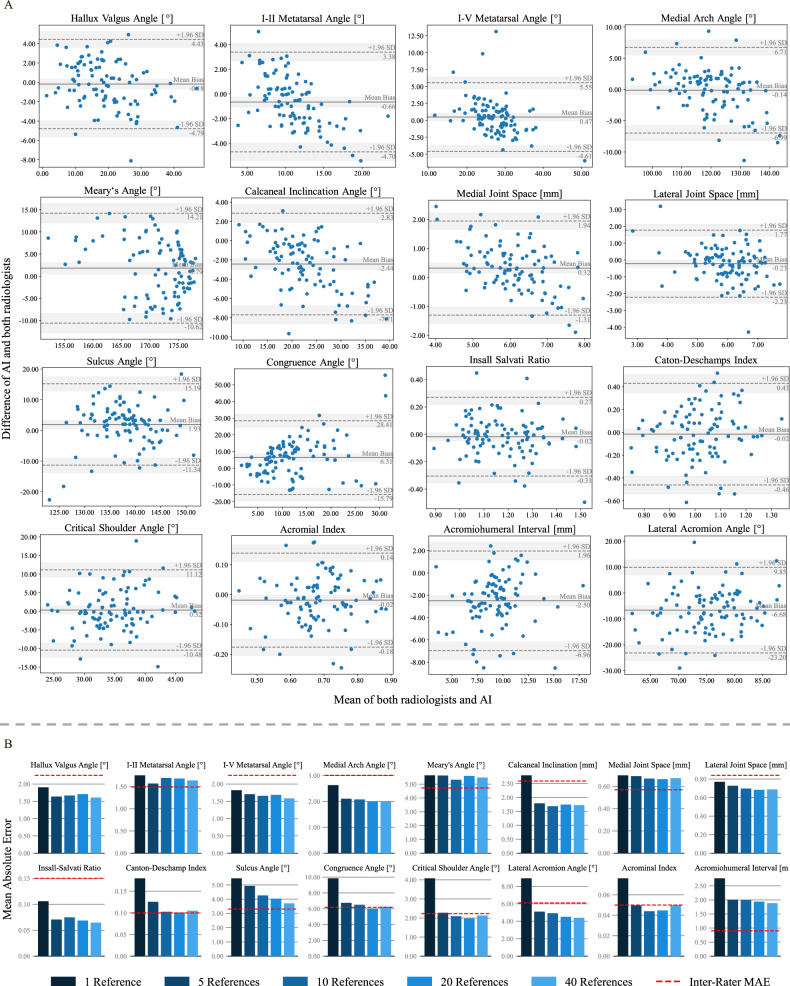
Agreement analysis and effect of reference image set size. **A** Bland–&&Altman plot. For each of the 16 morphometric measures, the difference between the automated measurement and the mean of both radiologists (*y*-axis) is plotted against their mean value (*x*-axis). The solid gray line denotes the mean bias, dashed lines indicate the 95% limits of agreement (± 1.96 SD). Bias is close to zero for most metrics except for calcaneal inclination angle, congruence angle, acromiohumeral interval, and lateral acromion angle. **B** Impact of multiple reference images. Bars show the MAE obtained with 1, 5, 10, 20, and 40 reference radiographs. The red dashed line indicates the inter-reader MAE between the two radiologists. Increasing the reference image pool lowered the MAE, and ≥ 5 reference images allowed the AI model to match or surpass inter-reader accuracy for nearly all measurements

These spatial patterns translated into the measurement domain as morphometric measurements derived from densely populated, low-error landmarks (e.g., I–II Metatarsal Angle, R1 vs AI: MAE = 1.81 ± 1.44°) exhibited the lowest MAEs, whereas measurements relying on the higher-error regions (e.g., Congruence Angle, R1 vs AI: MAE = 8.65 ± 8.42°) showed larger deviations from manual reference values (Supplementary Table S2). For several metrics, particularly those related to the foot and selected knee measurements, the AI model’s accuracy approached or exceeded that of both readers. However, other knee metrics (e.g., Congruence Angle) and most shoulder measurements showed larger deviations from manual reference values (Supplementary Table S2). Figure 4A presents the corresponding Bland–Altman plots. Overall, bias was minimal and evenly distributed across the measurement range, indicating no proportional error. Notable systematic offsets appeared only for the Congruence angle (positive) and the Lateral Acromion angle (negative). A few outliers were noted, most prominently affecting the Congruence angle. Importantly, the discrepancy between the AI model and both radiologists did not depend on the size of the measurement, as both showed comparable differences.

The impact of multiple reference images on accuracy was notable. Overall, landmark matching MAE decreased with a larger reference image pool, and when 40 reference images were used, the overall landmark matching MAE was reduced to 2.15 ± 2.38 mm (foot: 1.79 ± 1.84 mm; knee: 2.61 ± 2.98 mm; shoulder: 2.49 ± 2.34 mm). Correspondingly, the error and variance were reduced across most landmarks when using 40 reference images (Supplementary Fig. S4).

These improvements again translated into the measurement domain, where more than five references enabled the AI model to match or surpass inter-reader MAE for nearly all measurements (Fig. 4B). With only a few exceptions in cases where the ICC between the two radiologists was already low, the AI model approached or exceeded the radiologists’ ICC values when provided with 40 references (Table 3). In general, the inclusion of multiple references improved both accuracy and agreement across most measurements as compared to a single reference (Supplementary Table S1).

**Table 3:** Inter-method and inter-reader comparisons for 40 reference images

Anatomic region	Projection	Morphometric measurement	R1 vs R2	R1 vs AI	R2 vs AI
Foot	AP	Hallux Valgus angle [°]	2.25 ± 1.66 0.96 [0.94, 0.97]	1.86 ± 1.52 0.96 [0.92, 0.98]	2.08 ± 1.82 0.95 [0.93, 0.97]
		I–II Metatarsal angle [°]	1.49 ± 1.27 0.90 [0.86, 0.93]	1.61 ± 1.44 0.84 [0.73, 0.90]	1.99 ± 1.44 0.80 [0.62, 0.89]
		I–V Metatarsal angle [°]	2.25 ± 2.80 0.84 [0.78, 0.89]	1.53 ± 1.26 0.94 [0.91, 0.96]	2.20 ± 3.05 0.80 [0.71, 0.86]
	Lateral	Medial arch angle [°]	3.82 ± 3.01 0.91 [0.85, 0.94]	2.63 ± 1.91 0.96 [0.94, 0.97]	3.03 ± 2.42 0.93 [0.89, 0.95]
		Meary’s angle [°]	4.73 ± 4.08 0.69 [0.44, 0.82]	7.18 ± 4.53 0.40 [0.20, 0.57]	4.34 ± 3.21 0.57 [0.42, 0.69]
		Calcaneal inclination [°]	1.61 ± 1.70 0.95 [0.93, 0.97]	2.26 ± 1.95 0.91 [0.71, 0.96]	2.00 ± 1.70 0.92 [0.88, 0.95]
Knee	AP	Medial joint space [mm]	0.57 ± 0.47 0.81 [0.70, 0.87]	0.81 ± 0.65 0.45 [0.28, 0.59]	0.64 ± 0.48 0.62 [0.48, 0.72]
		Lateral joint space [mm]	0.84 ± 0.88 0.56 [0.37, 0.69]	0.82 ± 0.77 0.34 [0.15, 0.50]	0.84 ± 0.73 0.45 [0.29, 0.60]
	Lateral	Insall–Salvati ratio	0.15 ± 0.11 0.55 [−0.01, 0.79]	0.06 ± 0.05 0.87 [0.78, 0.92]	0.12 ± 0.11 0.58 [0.15, 0.78]
		Caton–Deschamps Index	0.10 ± 0.09 0.69 [0.46, 0.81]	0.12 ± 0.10 0.41 [0.24, 0.56]	0.11 ± 0.10 0.38 [0.20, 0.53]
	Axial	Sulcus angle [°]	3.31 ± 2.24 0.82 [0.67, 0.89]	3.27 ± 3.02 0.69 [0.54, 0.79]	4.55 ± 3.60 0.55 [0.21, 0.74]
		Congruence angle [°]	6.18 ± 4.96 0.50 [0.34, 0.63]	5.42 ± 4.31 0.28 [0.10, 0.45]	8.45 ± 6.94 0.02 [-0.17, 0.21]
Shoulder	AP	Critical shoulder angle [°]	2.23 ± 1.79 0.86 [0.76, 0.92]	2.23 ± 2.77 0.73 [0.57, 0.82]	2.58 ± 3.22 0.66 [0.53, 0.75]
		Lateral acromion angle [°]	5.44 ± 4.13 0.57 [0.43, 0.69]	3.90 ± 3.44 0.56 [0.40, 0.68]	5.70 ± 4.52 0.36 [0.18, 0.52]
		Acromial index	0.05 ± 0.22 0.27 [0.07, 0.44]	0.04 ± 0.05 0.73 [0.50, 0.84]	0.06 ± 0.23 0.19 [0.00, 0.37]
		Acromiohumeral interval [mm]	0.93 ± 1.25 0.81 [0.73, 0.87]	1.93 ± 1.62 0.63 [0.34, 0.78]	2.07 ± 1.62 0.62 [0.22, 0.88]

Morphometric measurements were compared between two radiologists (R1, R2) and the AI model (AI) in the routine cohort. Results are presented as MAE (MAE ± SD) and intraclass correlation coefficient (ICC [95% CI]). Units are indicated in square brackets

*AP* anteroposterior, *MAE* mean absolute error, *SD* standard deviation, *CI* confidence interval, *ICC* intraclass correlation coefficient

### Accuracy and precision of landmark matching and derived measurements on the challenging cohort

**Fig. 5:**
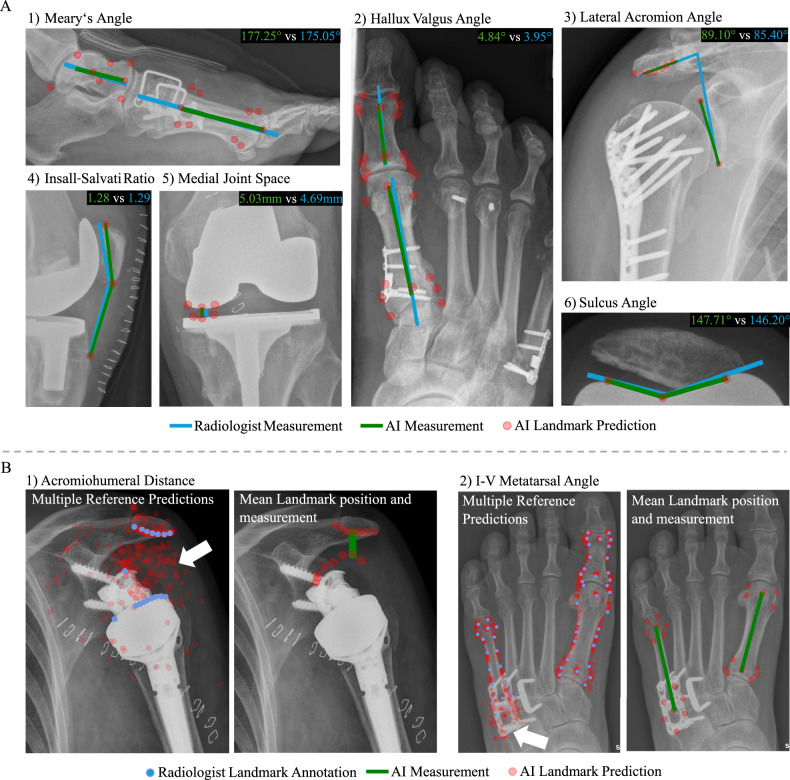
Examples of automated morphometric measurements in challenging radiographs. **A** Representative cases from the challenging cohort illustrates agreement between automated landmark predictions (red dots), subsequent automated measurements (green lines), and radiologist-provided manual reference measurements (blue lines). Numbers in each panel report AI vs radiologist values for the respective measurements. Examples span different anatomic regions and morphometric measurements, including (1) Meary’s angle, (2) Hallux Valgus angle, (3) lateral acromion angle, (4) Insall–Salvati ratio, (5) medial joint space, and (6) sulcus angle. Despite the presence of metal implants, automated measurements closely tracked those of radiologists in these cases. **B** Two representative cases that illustrate failures in landmark prediction (white block arrow). For each pair, the left image displays landmark predictions from multiple references (red dots) alongside radiologist annotations (blue dots), while the right image shows the averaged landmark positions (red dots) and resulting automated measurements (green lines). Shown are (1) the Acromiohumeral distance, where the presence of a reverse shoulder arthroplasty, skin staples, and off-center projection prevents correct landmark localization, and (2) the I–V metatarsal angle, where arthrodesis bone staples along the tarsometatarsal articulation bring about scattered landmark predictions, yet still allow the accurate identification of the fifth metatarsal after averaging

The challenging cohort was designed to identify limitations of our framework, acknowledging that automated measurements may not always be clinically feasible in such cases. All results were obtained using the routine reference set. Example measurements and failure cases are visualized in Fig. 5. Landmark matching (Supplementary Table S3) and morphometric measurements (Supplementary Table S4) showed mixed results in the presence of orthopedic hardware. It remained stable on some measures, such as the Hallux Valgus angle, whereas other measures were less stable, for example, the Meary’s angle (MAE + 4° in the challenging cohort vs the routine cohort) or the Congruence Angle (MAE + 6°). Consistent with the findings in the routine cohort, the use of multiple references improved accuracy for several measurements in the challenging cohort (Supplementary Table S4). ICC values increased correspondingly for most measurements, yet a subset of metrics retained near-zero or negative ICC values even with 40 references, indicating that these measurements cannot be considered clinically reliable in the presence of orthopedic implants without further improvement or additional quality control.

### Model visualizations

**Fig. 6:**
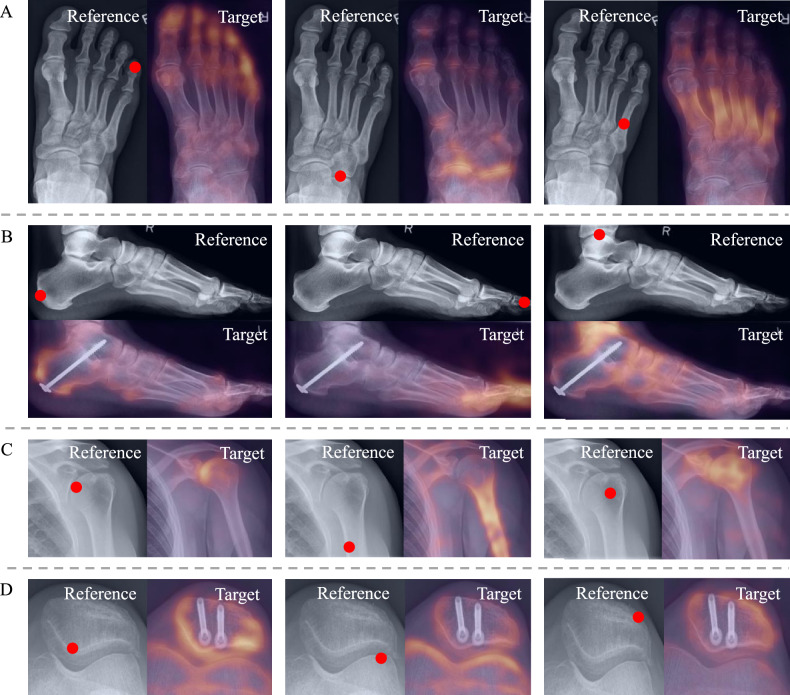
Feature-similarity maps reveal anatomy-aware matching and implant suppression. Each pair shows a reference radiograph (left/top) with red dots visualizing the queried points (not necessarily landmarks) and the corresponding target radiograph overlaid with a heatmap (right/bottom). The AI framework extracts image features across multiple scales, which, alongside positional data, are used to perform dense matching. The heatmap visualizes similarity between the feature vectors of the query point and all potential target pixels, computed as the Euclidean distance in the embedding space, with warmer colors indicating higher similarity. Despite being pretrained only on natural images, the framework consistently attends to anatomically corresponding structures across the projections ((**A**) foot AP, (**B**) foot lateral, (**C**) shoulder AP, and (**D**) axial-patellar view of the knee) while down-weighting metallic hardware (**B**, **D**). Consequently, the model relies on spatial and structural cues by integrating texture-based and semantic correlations

To interpret the framework’s internal representations, heatmaps were generated to illustrate the similarity between the internal feature representation of a query point in the reference image and the internal feature representations at all positions in the target image. The generated feature-similarity maps (Fig. 6) indicated that the network formed anatomy-aware correspondences, as the model highlighted queried points on reference images at corresponding locations in the target images. Overall, the integration of location and texture information, as well as semantic correlations, produced sound anatomic correspondences, both spatially and structurally, while non-anatomic image elements, such as metallic implants, were down-weighted. These patterns were stable across different anatomic regions and projections.

### Time demand and practical usability

The time required for landmark matching was evaluated using 100 runs. Matching one reference radiograph to one unseen target radiograph was substantially faster on a dedicated GPU machine (1.3 ± 0.4 s) compared to processing on a common consumer-grade notebook (89.4 ± 2.4 s) (see system specifications in the “Materials and methods”). In multi-reference setups, time demand increased approximately linearly with the number of reference images, as each reference was mapped individually to the target radiograph. Consequently, the time demand for those setups can be straightforwardly extrapolated by multiplying by the number of references. The automated framework had stable runtimes that were independent of the number of landmarks and the complexity of measurements.

Regarding annotation effort, the time required to place individual landmarks depends on the rater’s expertise with software and anatomy, and on image quality. In our experience, annotation took approximately 2–5 s per landmark, with additional overhead for software handling (e.g., opening and saving files). Because annotation speed varied considerably as familiarity with the workflow increased, formal timing was not recorded. Total annotation time scales with the number of landmarks required per projection (Table 2) and the number of reference radiographs used.

## Discussion

Current AI tools for quantitative morphometry in radiography must be trained in a task-specific manner on fully annotated datasets [10–20], which are challenging to create [3] and often exclude atypical cases, including metallic implants [12, 15–20]. In contrast, our generalist, minimal-setup, anatomy-agnostic, AI-based framework reframes landmarking and morphometry as dense matching between a single or a few annotated reference images and unseen target radiographs. Without anatomy-specific training or segmentation, the method produced accurate landmark predictions and morphometric measurements on an unfiltered series of patients from our clinical routine with standard foot, knee, and shoulder radiographs. Errors generally tracked inter-reader variability and improved with larger reference sets. In the presence of implants, several measurements remained comparable, whereas others (e.g., Meary’s and Congruence angles) deteriorated and may require additional quality control. Feature-similarity maps further suggested anatomy-aware correspondence while down-weighting metallic implants, which supports the clinical robustness and usability of this approach.

In contrast to other studies that often defined strict inclusion criteria, we intentionally assembled study cohorts from our routine clinical care without any patient-related exclusions (beyond age and incomplete series) to reflect real-world conditions. Although differing inclusion criteria may complicate head-to-head comparisons and introduce bias, we consider two publications relevant for benchmarking. On foot radiographs, our approach outperformed a landmark-prediction CNN trained specifically for the task [19] (e.g., MAE of Hallux Valgus Angle: 1.90° vs 4.14°) and approached the accuracy of a commercial solution [20] (1.90° vs 1.19°). With multiple references, performance converged toward that of the specialized commercial solution across multiple measures. We therefore consider our model competitive on routine data, while offering greater flexibility and versatility because it is (i) not limited to a fixed set of landmarks or a single anatomy, (ii) tested on challenging radiographs that are otherwise excluded, and (iii) independent of anatomy-specific training and extensive manual annotation.

Because new morphometric measurements can be added by defining landmarks on a handful of reference images, the framework can be rapidly adapted to additional projections or anatomic regions. It can accommodate radiologist- or institution-specific preferences and is well-suited for out-of-distribution radiographs (e.g., following the introduction of new surgical methods or devices). This combination of flexibility and versatility provides reproducible morphometric measurements in routine workflows and constitutes a pragmatic path to standardization, including in challenging clinical situations not foreseen at the time of model development.

In practice, the processing pipeline could be integrated into the PACS to automatically derive all relevant measurements immediately after image acquisition. This integration would substantially reduce turnaround time and provide the reporting radiologist with concurrent AI outputs, which could improve diagnostic efficiency substantially [30]. Even on-demand processing remains feasible, as users can balance accuracy and runtime by adjusting the number of reference images used.

Some limitations need to be mentioned. Performance depends on the quality and representativeness of the reference set, and unusual anatomy, superposition, or inaccurate annotations can degrade matching. Multiple references mitigated this issue, yet some metrics, such as Meary’s angle, remained error-prone, likely because they rely on structures with frequent overlap. Additionally, rescaling all images to 560 × 560 pixels during encoding may reduce spatial precision, particularly for fine anatomic structures, although upsampling to the original resolution before landmark matching partially mitigates this effect. Predicting robust reliability flags could help make this limitation more practical to manage, and incorporating additional anatomic priors, for example, through statistical shape models [31], may improve robustness, yet at the cost of higher computational load. Moreover, reference images were selected from within the same institutional dataset, which may introduce bias by favoring references that are highly representative of the evaluation cohort. Therefore, external generalizability to unseen institutions and acquisition protocols remains to be established, and future work should evaluate fixed a priori reference sets and external multi-center data. Moreover, our single-center, retrospective design limits the generalizability of our findings. However, the matching model was pretrained on natural, non-medical images and still demonstrated strong performance in the medical domain, despite the considerable domain gap. We therefore anticipate that this capability will extend to other institutions, scanner types, and radiographic views. Nonetheless, future studies should focus on multicenter validation, additional projections, and pediatric cohorts to confirm the model’s effective generalizability. On the other hand, remaining systematic offsets may reflect a domain shift of the pretrained backbone. Self-supervised fine-tuning on unlabeled radiographs (domain adaptation) is a promising strategy to better align the model with anatomy and potentially reduce such biases [32].

In conclusion, the presented generalist and training-free dense-matching strategy delivers morphometric measurements across diverse musculoskeletal radiographs with accuracy often comparable to that of trained radiologists and specialized tools. Minimal upfront annotation and straightforward extension to new measurements, anatomic regions, and projections make this approach well-suited for real-world deployment on appropriate GPU hardware, where robustness and standardization are as valuable as peak performance on narrowly defined patient cohorts.

## Supplementary information

ELECTRONIC SUPPLEMENTARY MATERIAL

## Data Availability

The software routines developed, validated, and implemented are publicly available on GitHub (https://github.com/TruhnLab/RadiographLandmarkMatching). Any other data for research purposes is available from the corresponding author upon reasonable request.

## Abbreviations

AI: Artificial intelligence
AP: Anteroposterior
CI: Confidence interval
CNN: Convolutional neural network
DINOv2: Distillation of knowledge with no labels (version 2)
MAE: Mean absolute error
PACS: Picture archiving and communication system
SD: Standard deviation
