# Supplementary material for: An artificial intelligence framework for universal landmark matching and morphometry in musculoskeletal radiography
Source: Eur Radiol. 2026 Apr 22;36(9):6936–49. doi: 10.1007/s00330-026-12555-y (PMC13451269; doi:10.1007/s00330-026-12555-y)
Supplement: Supplementary file 1 [file 330_2026_12555_MOESM1_ESM.pdf]

An Artificial Intelligence Framework for Universal Landmark Matching and Morphometry in Musculoskeletal Radiography

ELECTRONIC SUPPLEMENTARY MATERIAL

Supplementary Tables

Supplementary Table S1. Landmark matching errors for measurement-specific landmark subsets in the routine cohort by anatomy and projection. Mean landmark errors are reported as mean ± SD [95% CI] for both the single-reference and 40-reference configurations. Values are the Euclidean distances (in mm) between automated landmark predictions and manual landmark annotations, averaged over the subset of landmarks used to compute each measurement. AP = anteroposterior, CI = confidence interval, SD = standard deviation.

Anatomic Region	Projection	Measurement	Mean landmark error [mm]	
			1 Reference	40 References
Foot	AP	Hallux Valgus Angle	1.77±1.51 [1.71;1.83]	1.30±1.18 [1.25;1.34]
		I-II Metatarsal Angle	1.90±1.69 [1.83;1.97]	1.54±1.41 [1.48;1.60]
		I-V Metatarsal Angle	2.07±1.78 [2.00;2.14]	1.51±1.33 [1.46;1.57]
	Lateral	Medial Arch Angle	2.86±2.45 [2.62;3.10]	2.41±1.82 [2.23;2.59]
		Meary's Angle	3.29±2.64 [3.17;3.42]	2.62±2.22 [2.52;2.73]
		Calcaneal Inclination	2.97±2.47 [2.72;3.21]	2.43±1.81 [2.25;2.61]
Knee	AP	Medial Joint Space	1.71±1.36 [1.60;1.82]	1.57±1.26 [1.47;1.67]
		Lateral Joint Space	1.77±1.24 [1.67;1.87]	1.39±1.04 [1.30;1.47]
	Lateral	Insall-Salvati Ratio	3.90±3.25 [3.53;4.27]	2.78±2.97 [2.44;3.12]
		Caton-Deschamps Index	2.20±1.65 [1.96;2.43]	1.17±0.90 [1.04;1.29]
	Axial	Sulcus Angle	1.87±1.56 [1.65;2.09]	1.72±1.46 [1.51;1.92]
		Congruence Angle	2.20±1.87 [1.99;2.42]	1.93±1.82 [1.72;2.14]
Shoulder	AP	Critical Shoulder Angle	3.52±2.75 [3.20;3.83]	2.37±2.18 [2.12;2.62]
		Lateral Acromion Angle	2.94±2.50 [2.81;3.07]	2.67±2.43 [2.46;2.89]
		Acromial Index	4.01±3.35 [3.71;4.30]	2.40±2.31 [2.28;2.52]
		Acromiohumeral Interval	3.30±2.71 [3.12;3.48]	2.59±2.55 [2.42;2.76]

Supplementary Table S2. Inter-method and inter-reader comparisons for one reference image. Morphometric measurements were compared between two radiologists (R1, R2) and the AI model (AI) in the routine cohort. Results are presented as mean absolute error (MAE ± SD) and intraclass correlation coefficient (ICC [95% CI]). Units are indicated in square brackets. AP = anteroposterior, MAE = mean absolute error, SD = standard deviation, CI = confidence interval, ICC = intraclass correlation coefficient.

Anatomic Region	Projection	Morphometric Measurement	R1 vs. R2	R1 vs. AI	R2 vs. AI
Foot	AP	Hallux Valgus Angle [°]	2.25±1.66<br>0.96 [0.94, 0.97]	2.10±1.55<br>0.96 [0.94, 0.97]	2.22±1.79<br>0.95 [0.93, 0.97]
		I-II Metatarsal Angle [°]	1.49±1.27<br>0.90 [0.86, 0.93]	1.81±1.31<br>0.83 [0.75, 0.88]	2.01±1.51<br>0.79 [0.68, 0.86]
		I-V Metatarsal Angle [°]	2.25±2.80<br>0.84 [0.78, 0.89]	1.84±1.44<br>0.92 [0.87, 0.94]	2.28±3.12<br>0.79 [0.70, 0.85]
	Lateral	Medial Arch Angle [°]	3.82±3.01<br>0.91 [0.85, 0.94]	3.47±2.54<br>0.93 [0.89, 0.95]	3.14±2.85<br>0.91 [0.87, 0.94]
		Meary's Angle [°]	4.73±4.08<br>0.69 [0.44, 0.82]	7.36±4.69<br>0.38 [0.18, 0.55]	4.45±3.24<br>0.56 [0.41, 0.68]
		Calcaneal Inclination [°]	2.59±1.87<br>0.95 [0.93, 0.97]	3.37±2.73<br>0.82 [0.34, 0.93]	2.69±2.34<br>0.86 [0.67, 0.93]
Knee	AP	Medial Joint Space [mm]	0.57±0.47<br>0.81 [0.70, 0.87]	0.84±0.69<br>0.49 [0.28, 0.64]	0.63±0.52<br>0.64 [0.50, 0.74]
		Lateral Joint Space [mm]	0.84±0.88<br>0.56 [0.37, 0.69]	0.84±0.81<br>0.38 [0.20, 0.54]	0.94±0.82<br>0.44 [0.24, 0.58]
	Lateral	Insall-Salvati Ratio	0.15±0.11<br>0.55 [-0.01, 0.79]	0.10±0.10<br>0.57 [0.40, 0.70]	0.15±0.14<br>0.31 [0.11, 0.49]
		Caton-Deschamps Index	0.10±0.09<br>0.69 [0.46, 0.81]	0.19±0.16<br>0.00 [-0.19, 0.20]	0.18±0.13<br>0.05 [-0.15, 0.25]
	Axial	Sulcus Angle [°]	3.31±2.24<br>0.82 [0.67, 0.89]	5.53±4.84<br>0.38 [0.20, 0.53]	5.83±4.37<br>0.45 [0.25, 0.61]
		Congruence Angle [°]	6.18±4.96<br>0.50 [0.34, 0.63]	8.65±8.42<br>0.14 [-0.03, 0.31]	11.71±9.09<br>0.06 [-0.10, 0.22]
Shoulder	AP	Critical Shoulder Angle [°]	2.23±1.79<br>0.86 [0.76, 0.92]	4.02±3.33<br>0.58 [0.43, 0.69]	4.41±4.29<br>0.46 [0.29, 0.60]
		Lateral Acromion Angle [°]	5.44±4.13<br>0.57 [0.43, 0.69]	8.16±5.69<br>0.28 [0.02, 0.49]	9.94±7.55<br>0.15 [-0.04, 0.33]
		Acromial Index	0.05±0.22<br>0.27 [0.07, 0.44]	0.07±0.05<br>0.64 [0.49, 0.75]	0.06±0.05<br>0.64 [0.50, 0.74]
		Acromiohumeral Interval [mm]	0.93±1.25<br>0.81 [0.73, 0.87]	2.63±1.93<br>0.45 [-0.03, 0.71]	2.91±2.23<br>0.36 [-0.04, 0.63]

Supplementary Table S3. Landmark matching error for measurement-specific landmark subsets in the challenging cohort by anatomy and projection. Mean landmark errors are reported as mean ± SD [95% CI] for both the single-reference and 40-reference configurations, using the routine reference set to ensure identical operating points. Values are the Euclidean distances (in mm) between automated landmark predictions and manual landmark annotations, averaged over the subset of landmarks used to compute each measurement. AP = anteroposterior, CI = confidence interval, SD = standard deviation.

Anatomic Region	Projection	Morphometric Measurement	Mean landmark error [mm]	
			1 Reference	40 References
Foot	AP	Hallux Valgus Angle	1.85±1.68 [1.74;1.95]	1.52±1.34 [1.44;1.61]
		I-II Metatarsal Angle	2.29±1.85 [2.17;2.41]	1.93±1.56 [1.83;2.03]
		I-V Metatarsal Angle	2.30±1.90 [2.18;2.42]	1.86±1.55 [1.76;1.96]
	Lateral	Medial Arch Angle	4.25±4.18 [3.59;4.92]	3.69±3.82 [3.08;4.29]
		Meary's Angle	4.39±4.13 [4.08;4.70]	3.86±3.67 [3.59;4.13]
		Calcaneal Inclination	3.91±3.31 [3.38;4.43]	3.46±2.89 [3.00;3.92]
Knee	AP	Medial Joint Space	4.12±6.66 [3.27;4.96]	4.13±7.11 [3.22;5.03]
		Lateral Joint Space	4.71±9.92 [3.45;5.98]	4.51±10.06 [3.23;5.79]
	Lateral	Insall-Salvati Ratio	5.52±5.59 [4.51;6.54]	3.38±2.80 [2.87;3.89]
		Caton-Deschamps Index	4.15±3.92 [3.27;5.03]	2.52±2.23 [2.02;3.02]
	Axial	Sulcus Angle	9.45±15.20 [6.04;12.85]	9.01±15.27 [5.59;12.43]
		Congruence Angle	10.22±15.55 [7.40;13.04]	10.30±16.42 [7.32;13.28]
Shoulder	AP	Critical Shoulder Angle	4.77±5.64 [3.75;5.80]	3.39±4.43 [2.58;4.19]
		Lateral Acromion Angle	3.89±4.89 [3.38;4.39]	2.73±3.19 [2.40;3.06]
		Acromial Index	5.25±5.52 [4.48;6.02]	4.05±4.68 [3.40;4.70]
		Acromiohumeral Interval	4.14±4.82 [3.75;4.52]	3.32±3.83 [3.01;3.63]

Supplementary Table S4. Intermethod comparisons for multiple reference images. Indicated are the mean absolute error (MAE) ± SD and intraclass correlation coefficient (ICC [95% CI]) between the junior radiologist and the AI model in the challenging cohort (orthopedic hardware) as a function of the number of reference radiographs. Units are indicated in square brackets. AP = anteroposterior, MAE = mean absolute error, SD = standard deviation, CI = confidence interval, ICC = intraclass correlation coefficient, Ref. = Reference(s).

Anatomic Region	Projection	Morphometric Measurement	1 Ref.	5 Ref.	40 Ref.
Foot	AP	Hallux Valgus Angle [°]	2.22±1.91<br>0.88 [0.78, 0.93]	2.03±1.72<br>0.90 [0.82, 0.95]	1.84±1.62<br>0.91 [0.84, 0.95]
		I-II Metatarsal Angle [°]	2.29±1.57<br>0.51 [0.25, 0.71]	2.13±1.53<br>0.53 [0.27, 0.72]	1.83±1.27<br>0.66 [0.41, 0.81]
		I-V Metatarsal Angle [°]	2.05±1.81<br>0.84 [0.73, 0.91]	2.07±1.69<br>0.86 [0.74, 0.92]	1.83±1.65<br>0.88 [0.77, 0.93]
	Lateral	Medial Arch Angle [°]	5.40±14.41<br>0.46 [0.17, 0.68]	5.46±14.59<br>0.45 [0.16, 0.67]	5.58±14.75<br>0.44 [0.15, 0.66]
		Meary's Angle [°]	8.58±25.01<br>0.08 [-0.23, 0.38]	9.15±25.47<br>0.04 [-0.26, 0.34]	8.83±25.58<br>0.04 [-0.27, 0.34]
		Calcaneal Inclination [°]	2.81±2.40<br>0.81 [0.60, 0.91]	2.31±1.89<br>0.88 [0.78, 0.93]	2.32±1.80<br>0.88 [0.71, 0.95]
Knee	AP	Medial Joint Space [mm]	1.51±1.95<br>0.30 [0.01, 0.55]	1.40±1.70<br>0.39 [0.10, 0.62]	1.40±1.69<br>0.42 [0.13, 0.64]
		Lateral Joint Space [mm]	2.69±2.61<br>0.29 [-0.02, 0.55]	1.68±1.47<br>0.37 [0.08, 0.60]	1.71±1.60<br>0.26 [-0.04, 0.52]
	Lateral	Insall-Salvati Ratio	0.32±1.31<br>-0.01 [-0.32, 0.30]	0.30±1.31<br>-0.01 [-0.32, 0.30]	0.30±1.30<br>0.00 [-0.31, 0.31]
		Caton-Deschamps Index	0.40±1.19<br>-0.06 [-0.36, 0.26]	0.34±1.17<br>-0.01 [-0.31, 0.30]	0.32±1.18<br>-0.01 [-0.32, 0.30]
	Axial	Sulcus Angle [°]	9.81±8.62<br>0.42 [0.13, 0.65]	6.16±8.00<br>0.53 [0.27, 0.72]	3.97±3.80<br>0.77 [0.59, 0.87]
		Congruence Angle [°]	11.29±10.00<br>0.38 [0.08, 0.62]	12.65±12.40<br>-0.04 [-0.36, 0.27]	14.34±11.82<br>-0.16 [-0.44, 0.16]
Shoulder	AP	Critical Shoulder Angle [°]	5.76±6.83<br>0.05 [-0.27, 0.36]	3.25±4.05<br>0.36 [0.06, 0.60]	2.06±2.30<br>0.63 [0.40, 0.79]
		Lateral Acromion Angle [°]	6.81±9.82<br>-0.00 [-0.31, 0.31]	6.15±6.56<br>0.07 [-0.24, 0.38]	3.57±3.41<br>0.47 [0.19, 0.68]
		Acromial Index	0.12±0.17<br>0.07 [-0.24, 0.36]	0.10±0.14<br>0.23 [-0.05, 0.49]	0.08±0.13<br>0.27 [-0.03, 0.52]
		Acromiohumeral Interval [mm]	4.02±3.96<br>0.54 [0.25, 0.74]	3.35±3.50<br>0.62 [0.32, 0.79]	3.10±3.54<br>0.57 [0.25, 0.77]

Supplementary Figures

Hallux Valgus Angle

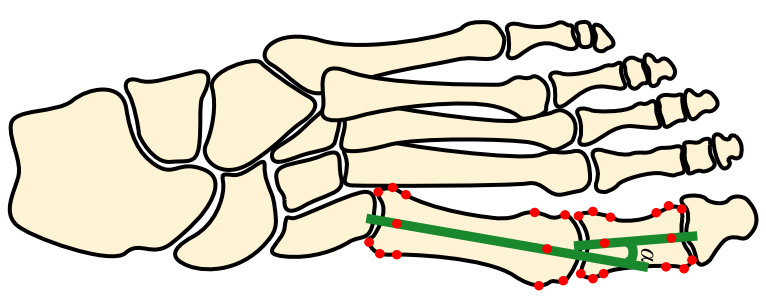

Angle created by the lines longitudinally bisecting the first metatarsal (12 landmarks) and the proximal phalanx (14 landmarks).

I-II Metatarsal Angle

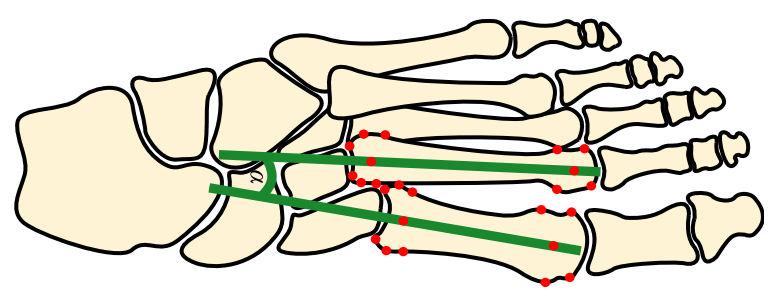

Angle created by the lines longitudinally bisecting the first metatarsal (12 landmarks) and the second metatarsal (12 landmarks).

I-V Metatarsal Angle

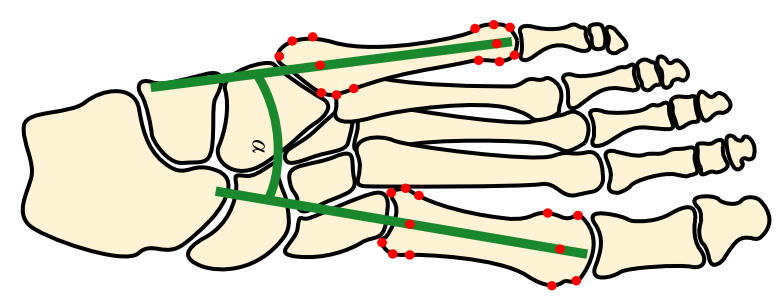

Angle created by the lines longitudinally bisecting the first metatarsal (12 landmarks) and the fifth metatarsal (14 landmarks).

Medial Arch Angle

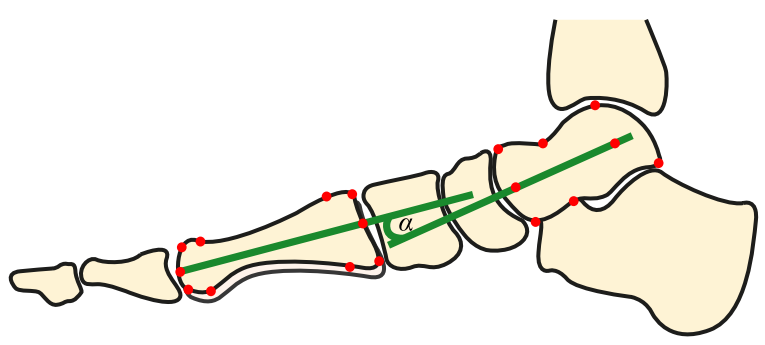

Angle defined by three points: the lowest point of the calcaneal tuberosity (1 landmark), the lowest point of the talonavicular joint (1 landmark), and the lowest point of the medial sesamoid (3 landmarks).

Meary's Angle

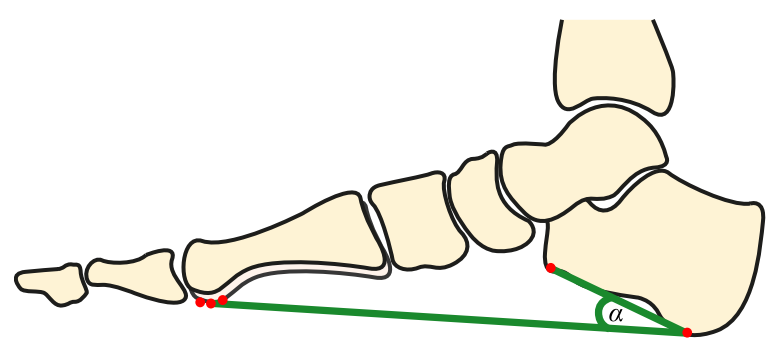

Angle created by the lines longitudinally bisecting the talus (8 landmarks) and the first metatarsal (10 landmarks).

Calcaneal Inclination

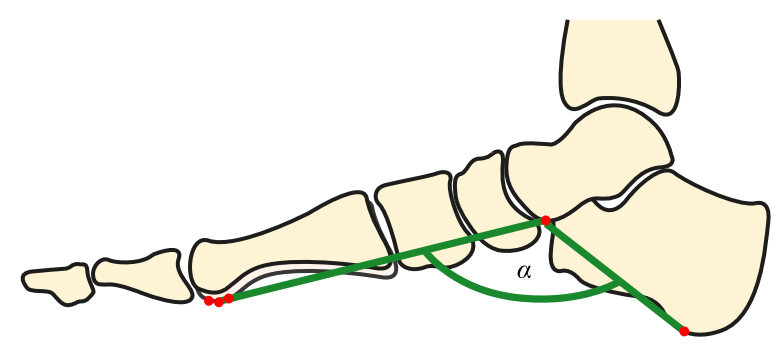

Angle defined by three points: the lowest point of the calcaneal tuberosity (1 landmark), the lowest point of the calcaneus at the calcaneocuboid joint (1 landmark), and the lowest point of the medial sesamoid (3 landmarks).

Supplementary Figure S1. Visualizations and explanations of the morphometric measures of the foot. For the foot, measurements were performed on the anterior-posterior (upper row) and lateral (lower row) views. Landmarks relevant to each specific measurement are highlighted in red, and constructed lines for subsequent quantification are shown in green. Angle measurements are denoted by α.

Insall-Salvati Ratio

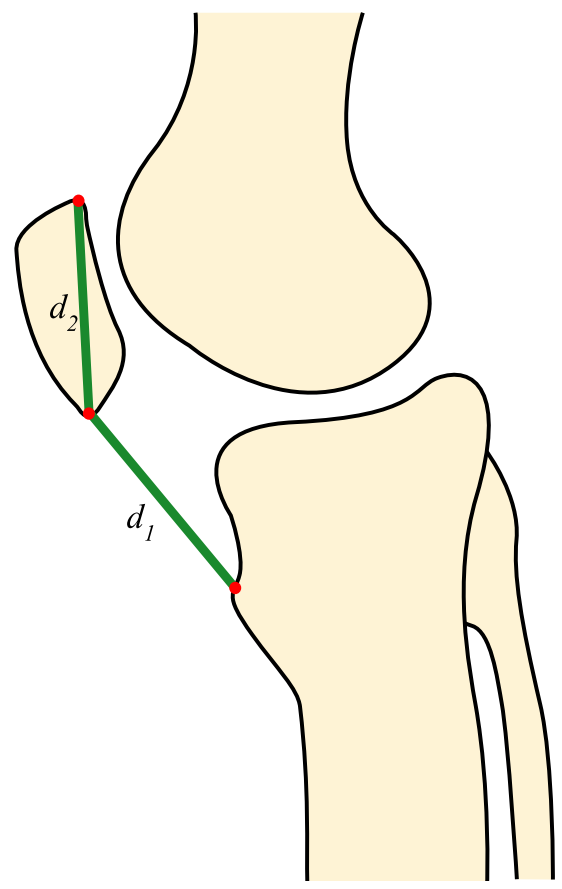

Ratio of the patellar tendon length (from patellar apex to tibial tuberosity, 2 landmarks) and the patella length (from patellar base to patellar apex, 2 landmarks).

Caton-Deschamp Index

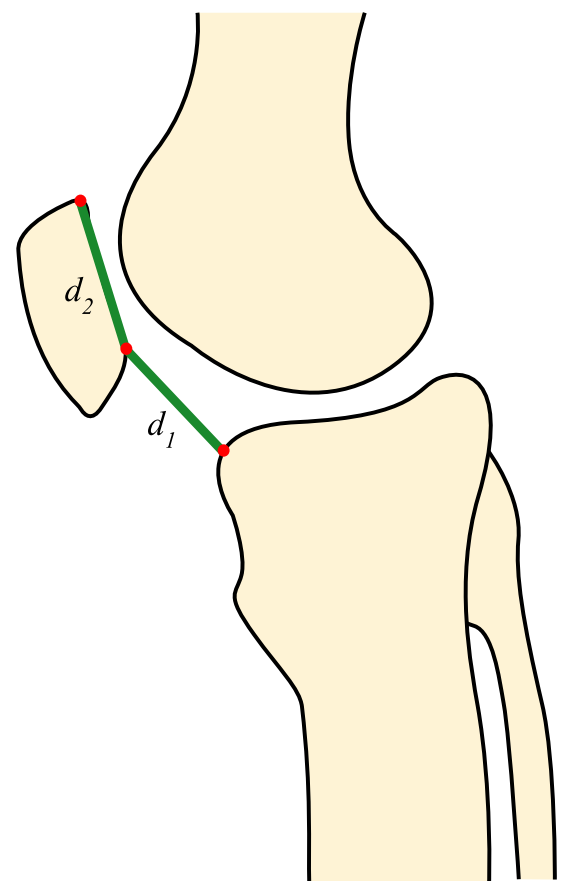

Ratio of the distance from the anterosuperior edge of the tibia to the lower edge of the patellar articular surface (2 landmarks) and the patellar articular surface length (2 landmarks).

Sulcus Angle

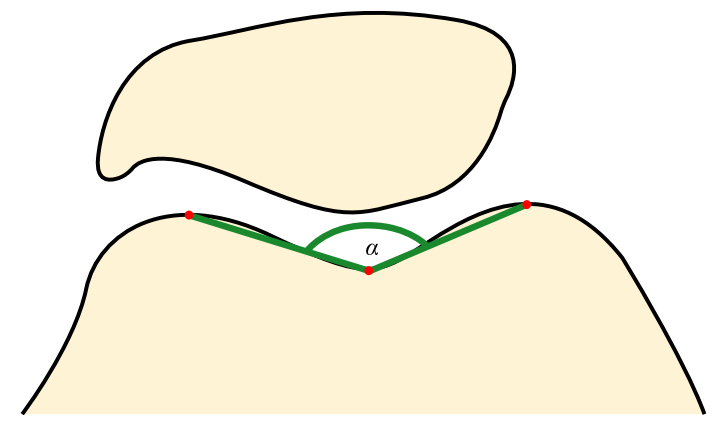

Angle defined by three points: highest points of both lateral and medial condyles (2 landmarks) and lowest point of the intercondylar sulcus (1 landmark).

Congruence Angle

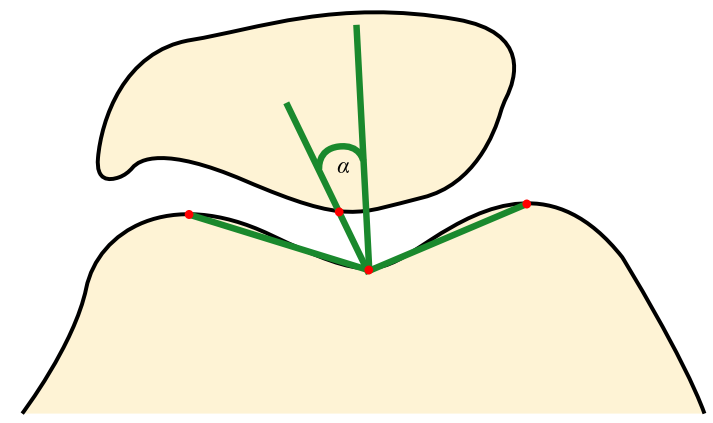

Angle created by the line connecting the lowest point of the patellar articular surface (1 landmark) and the lowest point of the intercondylar sulcus (1 landmark) with the line bisecting the sulcus angle (3 landmarks).

Lateral Joint Space

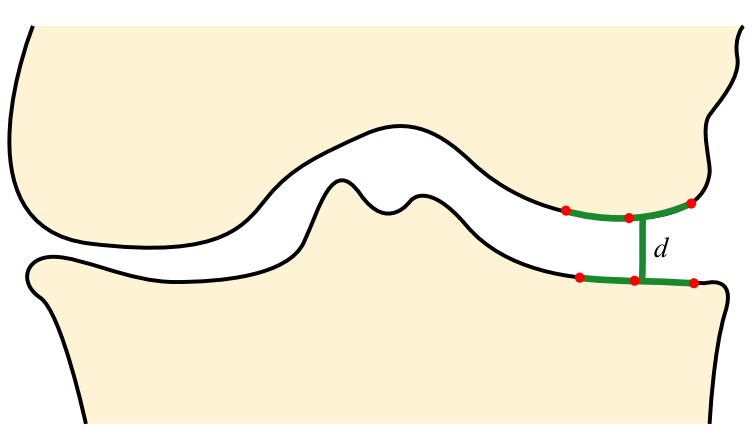

Smallest distance between the lateral femoral condyle (3 landmarks) and the lateral tibial plateau (3 landmarks).

Medial Joint Space

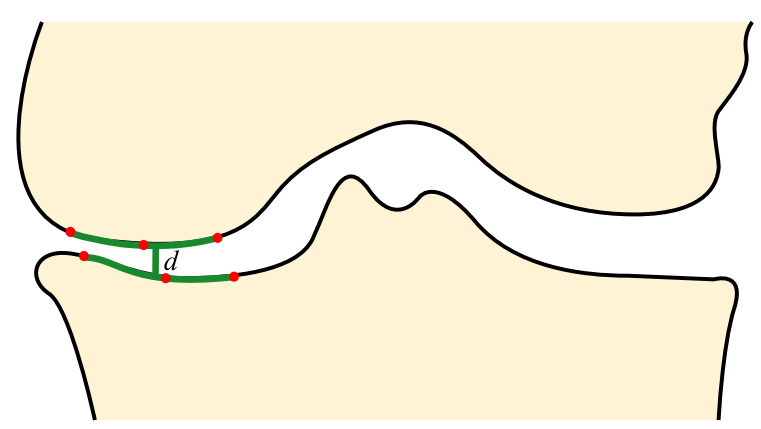

Smallest distance between the medial femoral condyle (3 landmarks) and the medial tibial plateau (3 landmarks).

Supplementary Figure S2. Visualizations and explanations of the morphometric measures of the knee. Knee measurements were performed on the lateral (upper left), anterior-posterior (lower left), and axial patellar views (right column). Landmarks are indicated in red and lines in green. Angle measurements are denoted by α, distances by *d*, and ratios by *d1/d2*.

Critical Shoulder Angle

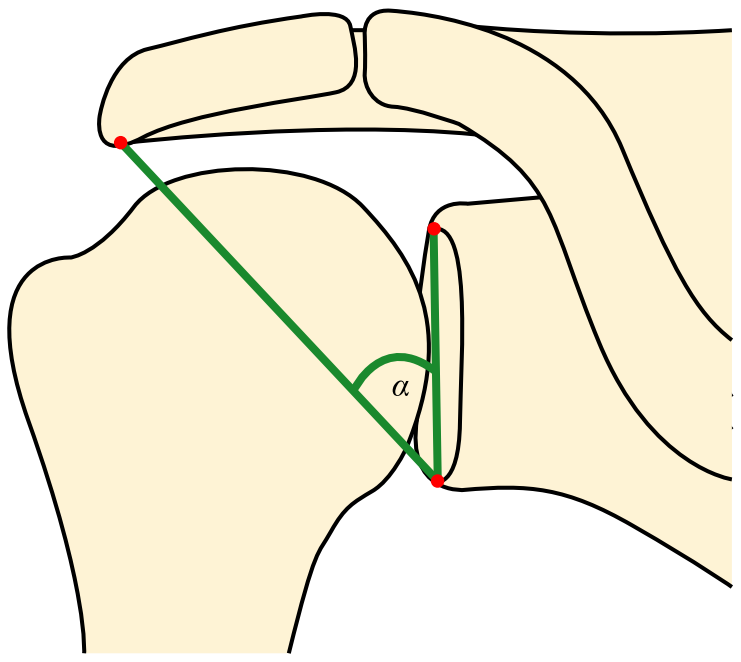

Angle defined by three points: superior and inferior edges of the glenoid fossa (2 landmarks) and the inferior lateral border of the acromion (1 landmark).

Lateral Acromion Angle

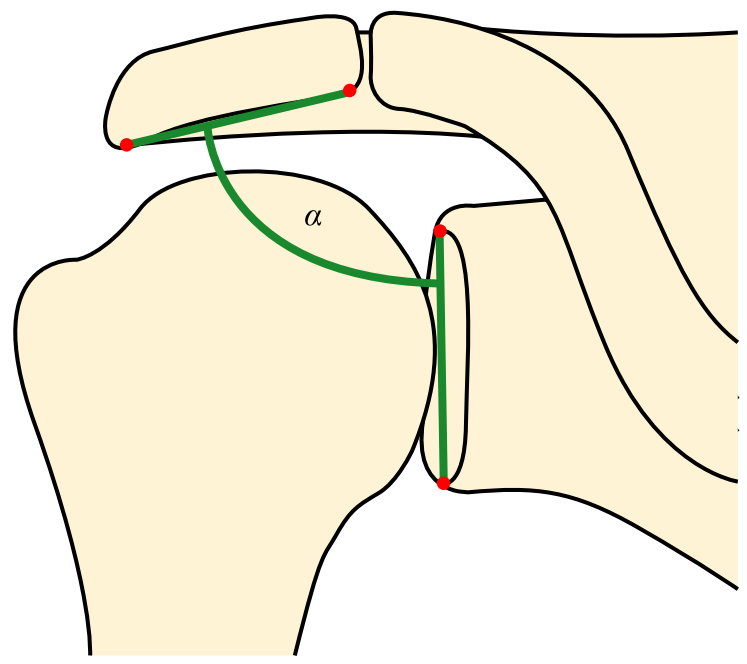

Angle created by the line along the acromion undersurface (2 landmarks) and the line across the superior and inferior edges of the glenoid fossa (2 landmarks).

Acromial Index

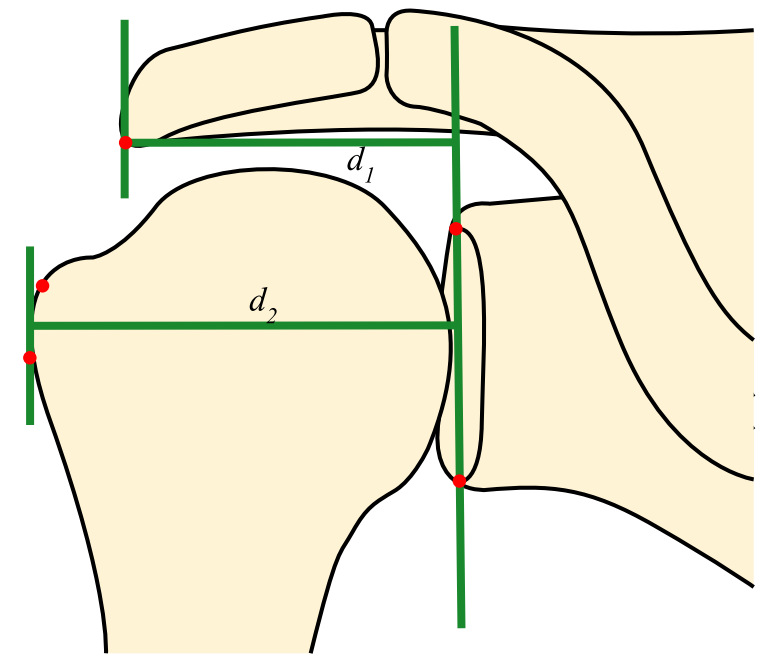

Ratio of the distances from glenoid fossa to lateral acromion and from glenoid fossa to lateral humeral head. Three parallel lines are constructed (1) across the superior and inferior edges of the glenoid fossa, (2) along the lateral border of the acromion, and (3) along the lateral border of the humeral head (4 landmarks). Two perpendicular lines are drawn and measured: d1, which corresponds to the distance between lines 1 and 2, and d2, which corresponds to the distance between lines 1 and 3.

Acromiohumeral Interval

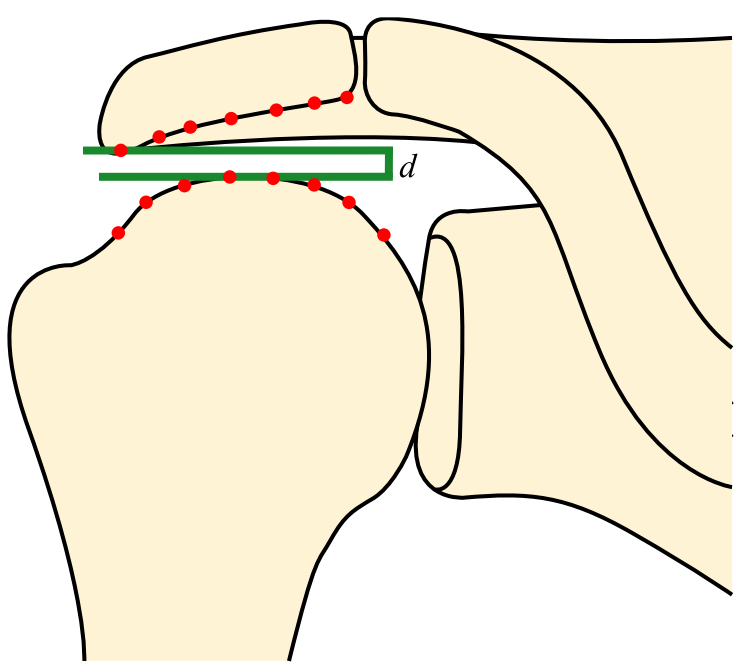

Distance between the lowest point of the acromion (7 landmarks) and the highest point of the humeral head (8 landmarks).

Supplementary Figure S3. Visualizations and explanations of the morphometric measures of the shoulder. Shoulder measurements were performed on the anterior-posterior view. Landmarks are indicated in red and lines in green. Angle measurements are denoted by α, distances by *d*, and ratios by *d1/d2*.

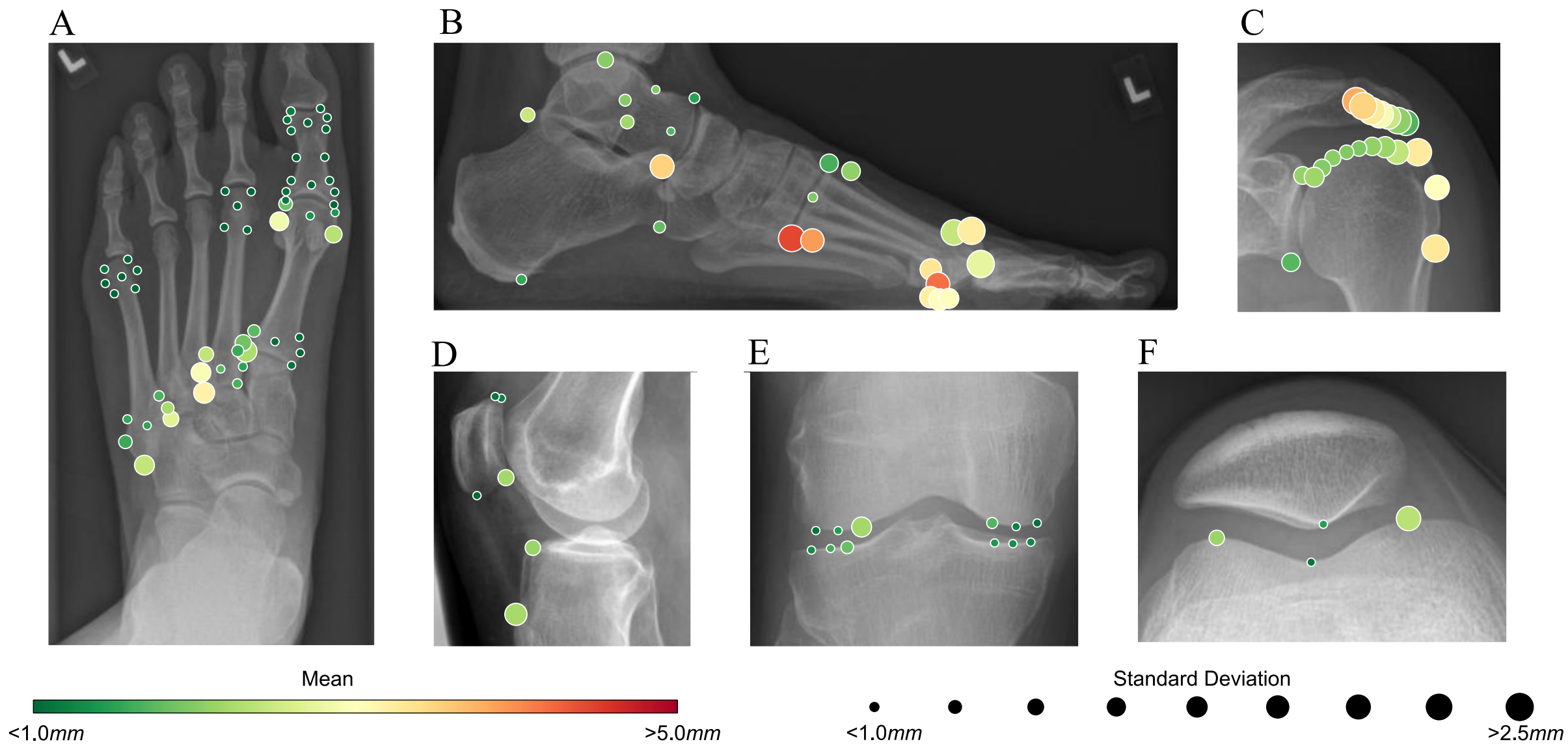

Supplementary Figure S4. Spatial distribution of landmark-matching errors across all projections with 40 reference images. Panels depict mean Euclidean error (colour-coded) and its standard deviation (dot size) for every landmark in the routine cohort, when predictions were aggregated from the top-40 automatically selected reference radiographs. The landmarks were used to derive the following morphometric measurements: (A) Foot anteroposterior (AP): Hallux Valgus Angle, I-II Metatarsal Angle, I-V Metatarsal Angle, (B) Foot lateral: Medial Arch Angle, Maery's Angle, Calcaneal Inclination, (C) Shoulder AP: Critical Shoulder Angle, Lateral Acromion Angle, Acromial Index, Acromiohumeral Interval, (D) Knee lateral: Insall-Salvati Ratio, Caton-Deschamp Index, (E) Knee AP: Medial and Lateral Joint Spaces, (F) Knee patellar axial: Sulcus Angle and Congruence Angle. More detailed descriptions of the morphometric measurements are presented in **Supplementary Figures S1-S3**.
